# Adiponectin Influences the Behavior of Stem Cells in Hormone-Resistant Breast Cancer

**DOI:** 10.3390/cells14040286

**Published:** 2025-02-15

**Authors:** Giuseppina Daniela Naimo, Martina Forestiero, Francesca Giordano, Adele Elisabetta Leonetti, Luca Gelsomino, Maria Luisa Panno, Sebastiano Andò, Loredana Mauro

**Affiliations:** 1 Department of Pharmacy Health and Nutritional Sciences, University of Calabria, 87036 Arcavacata di Rende, CS, Italy; giuseppinadaniela.naimo@unical.it (G.D.N.); martina.forestiero@unical.it (M.F.); francesca.giordano@unical.it (F.G.); adeleelisabetta.leonetti@unical.it (A.E.L.); luca.gelsomino@unical.it (L.G.); mluisa.panno@unical.it (M.L.P.); 2 Centro Sanitario, University of Calabria, 87036 Arcavacata di Rende, CS, Italy

**Keywords:** adiponectin, breast cancer, endocrine resistance, tamoxifen, cancer stem cells, apoptosis

## Abstract

In the breast tumor microenvironment (TME), adipocytes exert a selective pressure on the behavior of breast cancer stem cells (BCSCs), which are involved in endocrine therapy resistance. In obesity, adipocytes secrete reduced levels of adiponectin, which promotes the growth and progression of ERα-positive breast cancer (BC). Here, we examined how low adiponectin levels affect the enrichment of the BCSC subpopulation and the mechanisms contributing to the maintenance of endocrine therapy resistance in BC. Flow cytometry, qRT-PCR, and Western blotting analysis were performed to assess stemness, the cell cycle, and apoptosis markers in MCF-7 wild-type (WT) and tamoxifen-resistant (TR) mammospheres. nLC-MS/MS was employed to profile and compare the proteome of BCSCs. Differentially expressed proteins were intersected with data from the Metacore^TM^ dataset. Our study demonstrated that adiponectin increased the percentage of CD44^+^/CD24^−^/ALDH1^+^ stem-like cells in TR MCF-7 mammospheres. Specifically, adiponectin contributed to the maintenance of BCSC bulk in TR MCF-7 cells through a slow cycling rate, supported by decreased levels of Cyclin D1 and Ki67 and increased p21 and p27 expression, and through escape from apoptosis, sustained by reduced ROS production and preserved maintenance of mitochondrial membrane potential. Our results provide new insights into the contribution of adiponectin to poor ERα-positive BC outcomes. Deeply understanding adiponectin’s role in stemness may disclose novel therapeutic approaches to treat hormone-resistant obese BC patients.

## 1. Introduction

The International Agency for Research on Cancer (IARC) estimated 2.3 million new cases of breast cancer in 2022. These represent 11.6% of all cancer cases worldwide, making breast cancer the most common malignancy in terms of incidence and mortality among women [1].

Approximately 70–80% of all breast cancers diagnosed express estrogen receptor (ER), which has a positive prognostic value [2]. In clinical practice, the initial treatment option for ER-positive patients is endocrine therapy. This approach aims to inhibit tumor growth by reducing estrogen stimulation [3]. It involves the use of selective estrogen receptor modulators, such as tamoxifen, selective estrogen receptor degraders, or inhibitors of the enzyme aromatase [4].

Despite the efficacy of anti-estrogen drugs in reducing mortality, approximately 15–20% of ER-positive breast cancers exhibit intrinsic resistance, while an additional 30–40% develop acquired resistance to treatment over time [5]. Among the main endocrine drugs, tamoxifen remains the primary and frontline therapeutic agent for the treatment of ER-positive breast cancers [2]. Thus, overcoming tamoxifen resistance is currently one of the major challenges in clinical practice.

Several mechanisms have been proposed in endocrine resistance, including loss of ERα expression, gain-of-function mutations in ERα, crosstalk between ERα signaling and other pathways, cell cycle deregulation, and heterogeneity of tumor cell population [6,7,8,9,10,11].

In particular, accumulating evidence has identified a subpopulation of undifferentiated cells within the tumor, called breast cancer stem cells (BCSCs), as responsible for resistance to anti-cancer therapies, recurrence, and metastasis [12,13,14]. These cells are able to divide asymmetrically in order to preserve their own pool and to generate tumor bulk, and they are also able to grow in low-adherence cell culture conditions [15,16,17]. Moreover, BCSCs survive oxidative stress and DNA damage due to their capacity to persist in a long-term quiescent G0 phase and their anti-apoptotic properties [18,19]. All this makes them critical players in maintaining the tumor and its malignant behavior, as well as in resistance to radiation, chemotherapy, and molecular target therapy [20,21].

The intricate interplay between autocrine signals, derived from tumor cells, and paracrine interactions, emerging from the tumor-associated stroma, has been demonstrated to be pivotal in the induction and maintenance of a stem-like state in cancer cells [22]. In breast cancer, there is compelling evidence that tumor microenvironment (TME) components such as adipocytes, stromal fibroblasts, immune cells, and endothelial cells sustain BCSC bulk through the secretion of several growth factors and cytokines [23]. In this context, adipocytes exert a considerable influence on the biological behavior of cancer cells, representing the most abundant cellular component of the breast TME [24,25,26]. Furthermore, numerous clinical studies have identified adipocytes as key mediators in the obesity–cancer relationship, with obese adipocytes exhibiting a dysfunctional secretion profile [24,27,28,29,30,31,32,33,34]. Among the adipokines released by adipose tissue, adiponectin is the main adipocyte-derived factor, and its levels are reduced in obese compared to normal-weight women [27].

Several epidemiological studies and clinical investigations have demonstrated an inverse correlation between adiponectin levels and the risk of developing breast cancer, as well as an association between lower adiponectin levels and more advanced stages of malignancy [35]. In addition, these studies highlighted a reduced disease-free survival and overall survival in breast cancer patients with low levels of adiponectin [36]. Recent in vitro and in vivo studies have contributed to elucidating the role of adiponectin in the growth and progression of ER-positive breast cancer, identifying this adipokine as a tumor-promoting growth factor [28,29,30,37,38,39,40].

Based on this evidence, the present study aimed to ascertain whether low adiponectin levels may promote the enrichment of BCSCs, which are known to fuel endocrine resistance and tumor relapse, in a tamoxifen-resistant breast cancer model, thereby contributing to tumor aggressiveness.

## 2. Materials and Methods

### 2.1. Cell Culture

The human breast cancer epithelial cell line MCF-7 (WT MCF-7) was purchased from the American Type Culture Collection (ATCC, Manassas, VA, USA). Cells were cultured in DMEM/F-12, supplemented with 10% fetal bovine serum (FBS), 200 mM L-glutamine, and 1% penicillin/streptomycin (Life Technologies, Monza, Italy). The tamoxifen-resistant (TR) MCF-7 cell line was obtained by long-term cultivation of parental cells with increasing concentrations of tamoxifen (TAM, 10^−6^ M 4-hydroxytamoxifen, Sigma-Aldrich, Merck, Milan, Italy), followed by chronic exposure to TAM at a concentration of 1 µM. The acquired resistance was periodically checked for the absence of TAM-dependent growth inhibition in comparison to the parental cells [41]. Cells were maintained at 37 °C in a humidified 5% CO_2_ atmosphere. For experimental purposes, cells were synchronized in phenol red-free and serum-free media for 24 h and then untreated (Control, C) or treated with globular adiponectin 5 μg/mL (A5, Prospect, Rehovot, Israel) in a phenol red-free medium containing 10% dextran charcoal-stripped FBS. All experiments were performed with mycoplasma-free cells.

### 2.2. Mammosphere Assay

WT and TR MCF-7 monolayer cells were pre-treated in adherence conditions with adiponectin 5 µg/mL for 48 h and then enzymatically disaggregated to obtain single-cell suspension. Single cells were plated in ultralow attachment plates at a density of 500 cells/cm^2^ in phenol red-free and serum-free DMEM/F12 supplemented with B-27 (Invitrogen, Life Technology, Milan, Italy) and 20 ng/mL Epidermal Growth Factor (EGF, Sigma-Aldrich, Merck, Milan, Italy). After 7 days, mammospheres ≥ 50 μm diameter (primary-generation mammospheres) were counted using a microscope (×40 magnification), collected, enzymatically dissociated, and plated at the same seeding density used in the primary generation to obtain secondary mammospheres. No additional treatments were added in the secondary generation. Mammosphere-forming efficiency (MFE) was calculated by dividing the number of mammospheres formed by the number of single cells seeded per well and expressed as a percentage. Mammosphere data are presented as the percentage of mammosphere formation in adiponectin-treated samples normalized to the percentage of mammosphere formation in control samples. Mammosphere self-renewal was calculated by dividing the number of secondary mammospheres formed by the number of primary-generation mammospheres [42].

### 2.3. Flow Cytometric Detection of CD44, CD24 and ALDH1 Expression

Secondary-generation mammospheres were digested using PBS containing 0.25% trypsin, 0.02% EDTA (Life Technology, Milan, Italy) to obtain a single-cell suspension. To detect CD44 and CD24 expression, cells were washed in PBS with 2.5% BSA and stained with FITC anti-human CD44 and PE anti-human CD24 (BD Biosciences, Milan, Italy) according to the supplier’s protocol. To detect ALDH1 expression, cells were incubated in ice-cold methanol for 10 min at −20 °C. Subsequently, the pellet was resuspended in 0.1% Triton X-100 and incubated for 15 min at Room Temperature (RT). Finally, cells were washed in PBS with 2.5% BSA and stained with FITC anti-human ALDH1A1 antibody (Santa Cruz Biotechnology, Milan, Italy), according to the manufacturer’s instructions. Flow cytometric analysis was performed with CytoFLEX flow cytometry (Beckman-Coulter, Milan, Italy) and acquisition data were obtained with CytExpert Beckman Coulter software (version number 2.4).

### 2.4. Total RNA Extraction, RT-PCR, and qRT-PCR Assay

Gene expression was evaluated by quantitative real-time reverse transcription PCR (qRT-PCR) using SYBR Green Universal PCR Master Mix (Thermo Fisher Scientific, Monza, Italy). 18S mRNA content was used to normalize each sample. The primers used are listed in Table 1.

### 2.5. Proteomic Analysis

Unless otherwise indicated, all chemicals used in the experiments were purchased from Sigma-Aldrich (St. Louis, MO, USA).

#### 2.5.1. Protein Digestion

A 20 µg aliquot of whole-cell extract obtained from secondary-generation mammospheres was diluted to a final volume of 32 µL with RIPA buffer. To achieve the reduction and alkylation of cysteines, 3.2 µL of 100 mM Dithiothreitol (DTT, 1 h incubation at 37 °C), 3.8 µL of 200 mM iodoacetamide (1 h incubation at 37 °C), and finally 0.6 µL (1 h incubation at 37 °C) of 100 mM DTT were added sequentially. Next, the Protein Aggregation Capture (PAC) protocol was used to digest half of each sample (10 µg) [43]. Briefly, for each sample, two sequential washes with 100 µL of 70% acetonitrile (ACN) were performed to equilibrate 5 µL (100 µg) of MagResyn Hydroxyl beads. Samples were then added to the magnetic microparticles, and protein precipitation was achieved by adding ACN to a final organic/water ratio of 70:30. The solution was incubated at 1100 rpm for 10 min. This was followed by a wash with 70% ethanol after three washes with 200 µL ACN. Finally, bead resuspension was carried out by adding 50 μL of 50 mM triethylammonium bicarbonate (TEAB), and trypsin (from porcine pancreas) was added to each sample at an E/S1/50 (200 ng). After overnight incubation (37 °C, 1100 rpm), the peptide solution was harvested and a single wash of the beads with 50 µL of 0.1% formic acid (FA) was performed to recover residual peptides.

#### 2.5.2. Nanoscale Liquid Chromatography Mass Spectrometry/Mass Spectrometry (nLC-MS/MS)

A Q-Exactive hybrid quadrupole-orbitrap mass spectrometer (Thermo Fisher Scientific) operating in positive ion mode, coupled with an Easy LC 1000 nanoscale liquid chromatography system (Thermo Fisher Scientific), was used to carry out a mass spectrometry (MS) analysis. The analytical column consisted of a 15 cm pulled silica capillary (75 µm i.d.) packed in-house with 3 µm C18 silica particles (Dr. Maisch). By applying a potential of 2000 V to the front end of the column through a tee piece, nanoelectrospray (nESI) was obtained. A binary gradient was used to obtain eluted peptides, with two mobile phases consisting of 2% ACN/01% FA (v:v) (mobile phase A) and 80% ACN/01% FA (mobile phase B). The flow rate was set to 230 nl/minutes. The mobile phase B ramped from 6% to 28% in 90 min, from 28% to 45% in 30 min, and from 45% to 100% in 8 min. It was then held at 100% for 10 min. The analytical column was equilibrated at 0% mobile phase B for 2 min. Data Independent Acquisition (DIA) included 20 windows with a full scan at 70,000 resolution (AGC target of 1e6 and maximum injection time of 50 ms) and 20 DIA scans at 35,000 resolution (AGC target of 1e6, maximum injection time of 120 ms and normalized collision energy of 25). Specifically, the isolation window was set to have 4 windows of 30 *m*/*z*, 13 windows of 20 *m*/*z*, and 3 windows of 50 *m*/*z*. The overlap was 1 *m*/*z*. The resulting *m*/*z* range was 370–900.

#### 2.5.3. Data Analysis and Results Reporting

The nLC-MS/MS data were searched using Spectronaut software v.17 against the human reference proteome (79,740 seq, Uniprot 11 October 2022) and a database of common contaminants (46 entries). Analysis settings were left as default, with only the following changes to the default parameters: precursor filtering was set to filter out all precursors identified in fewer than 3 runs for each comparison. Missing values were imputed using a run-wise imputation strategy. The maximum number of precursors used for quantification was set to 5. For quantification, only peptides shared between proteins of the same protein group (protein group specific) were taken into account. Perseus v.2.06.0 was then loaded with the resulting report for statistical analysis. For all comparisons, the analysis was performed as follows: Protein Quantities calculated by the Spectronaut software were log2 transformed, the matrix was then filtered to retain only protein groups quantified in at least 3 samples of a group. Then, differentially expressed proteins (DEPs) were identified by applying a two-sided *t*-test (*p*-value < 0.01). The mass spectrometry proteomics data have been deposited to the ProteomeXchange Consortium via the PRIDE partner repository with the dataset identifier PXD059964.

### 2.6. MetaCore^TM^ Analysis

For network and enrichment analysis, we used MetaCore^TM^ (GeneGo, Clarivate Analytics, London, UK) software, a knowledge database suitable for pathway analysis of experimental data and gene lists. To explore the biological significance of the DEPs obtained from the nLC-MS/MS analysis, enrichment Gene Ontology (GO) annotations were generated. The algorithm “analyze networks” and the high-trust interactions for Homo sapiens species were applied to connect the list of associated network-objects with other nodes in the human interactome. MetaCore^TM^ matches input genes to more than one object if the gene itself is associated with other gene products or is part of a molecular complex. The obtained networks were mapped to a GO process, linking them to biological function. The comparison was carried out using a fold change (log2-FC) > 1 and an adjusted *p*-value of <0.05, expressed in -log(*p*-value) and ranked by statistical significance. MetaCore^TM^ legend is available at the following link: https://portal.genego.com/legends/MetaCoreQuickReferenceGuide.pdf.

### 2.7. Cell Cycle Analysis

Disaggregated mammospheres were pelleted, washed with PBS, and fixed in 50% methanol overnight (ON) at −20 °C. Then, cells were stained with a solution containing 50 μg/mL propidium iodide (PI), 20 U/mL RNase, and 0.1% Triton X-100. Cell cycle phases were estimated as a percentage of a total of 10.000 events. DNA content was measured using CytoFLEX flow cytometry (Beckman-Coulter, Milan, Italy) and data were acquired using CytExpert Beckman Coulter software [44].

### 2.8. Western Blotting

Equal amounts of total protein were resolved on SDS–polyacrylamide gels and transferred to a nitrocellulose membrane by electro-blotting. The membranes were blocked in 5% non-fat dry milk and probed ON at 4 °C with primary antibodies. The antigen–antibody complex was detected by incubation of the membranes for 1 h at RT with a peroxidase-coupled anti-IgG antibody and revealed using an enhanced chemiluminescence system (ECL, Santa Cruz Biotechnology, Milan, Italy). Images were acquired by using IBright (ThermoFisher Scientifics, Milan, Italy) and analyzed by ImageJ software.

### 2.9. Proliferation Assay

Cells isolated from secondary-generation mammospheres were seeded in flat-bottom 96-well plates (10 × 10^3^ cells/well). After 24 h, cells were treated with adiponectin for 48 h in a phenol red-free medium containing 10% dextran charcoal-stripped FBS. Cell viability was assessed using a FluoReporter Blue Fluorometric dsDNA Quantitation Kit (ThermoFisher Scientific, Monza, Italy) according to the manufacturer’s protocol.

### 2.10. Annexin V/PI Assay

Early apoptotic events were evaluated using a Dead Cell Apoptosis Kit with Annexin V FITC and propidium iodide for flow cytometry (Invitrogen, Life technology, Milan, Italy). Cells obtained from secondary-generation mammospheres were stained with FITC-conjugated Annexin V and PI according to the supplier’s instructions. Samples were analyzed by CytoFLEX flow cytometry (Beckman-Coulter, Milan, Italy) and data were acquired using CytExpert Beckman Coulter software [44].

### 2.11. Reactive Oxygen Species (ROS) Assessment

ROS production was measured using the chloromethyl derivative of 2′,7′-dichlorodihydrofluorescein diacetate CM-H2DCFDA (Thermo Fisher Scientific, Monza, Italy). Secondary mammosphere-derived cells were collected and incubated with 10 µM CM-H2DCFDA following the manufacturer’s recommendations. ROS levels were estimated by using the mean fluorescent intensity of the viable cell population. The results were obtained with CytoFLEX flow cytometry (Beckman-Coulter, Milan, Italy) and analyzed using CytExpert Beckman Coulter software [44].

### 2.12. Mitochondrial Membrane Potential Assay

Cells obtained from secondary-generation mammospheres were collected and incubated with 10 µM MitoTracker Orange Chloromethyltetramethylrosamine (CMTMRos, Thermo Fisher Scientific, Monza, Italy) following the manufacturer’s instructions. Changes in mitochondrial membrane potential were detected by CytoFLEX flow cytometry (Beckman-Coulter, Milan, Italy) and analyzed using CytExpert Beckman Coulter software [44].

### 2.13. Statistical Analysis

Each data point represents the mean ± S.D. of at least 3 independent experiments. Data were analyzed by Student’s T test using the Prism 7.0 (GraphPad Software) software program. *p* < 0.05 was considered statistically significant.

## 3. Results

### 3.1. Effects of Adiponectin on Stem Subpopulation Enrichment in TR MCF-7 Mammospheres

**Figure 1:**
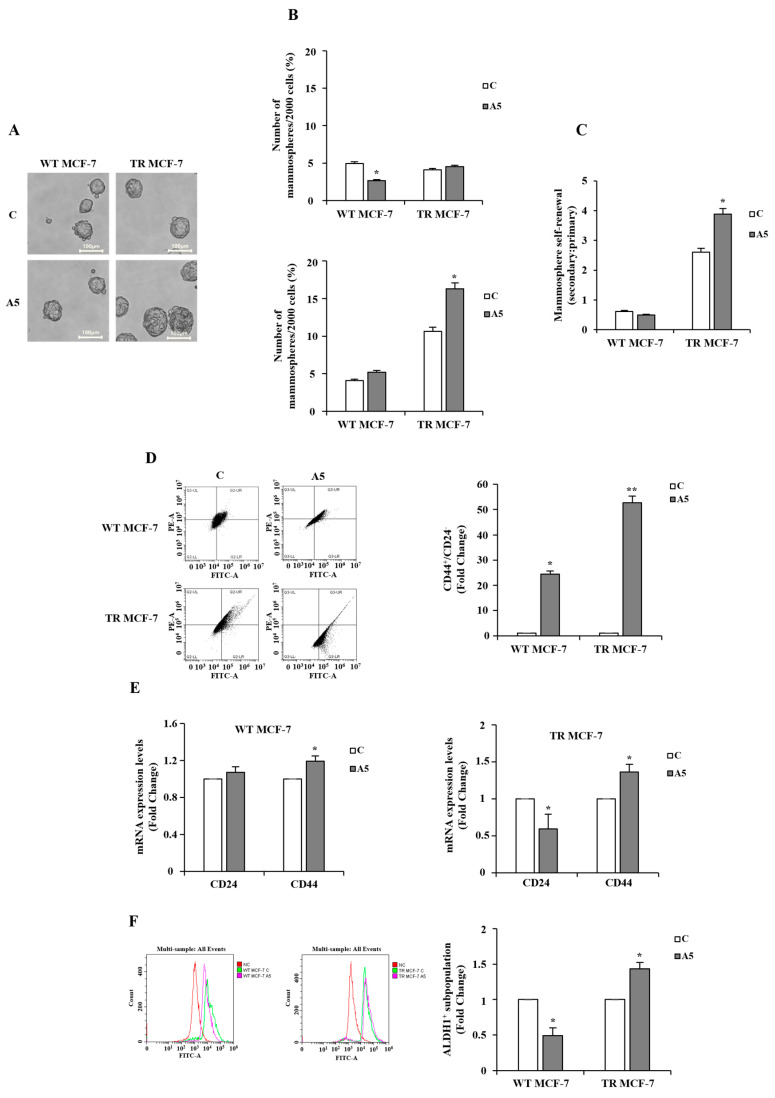
Breast cancer stem cell enrichment in WT and TR MCF-7 mammospheres. (**A**) Representative bright-field images of secondary WT and TR MCF-7 mammospheres in the absence (control, C) or presence of adiponectin 5 μg/mL (A5) acquired using a sCMEX-3 microscope camera (Euromex, Spain) and ImageFocus Alpha software. Scale bar = 100 μm. (**B**) MFE evaluated in WT and TR MCF-7 primary (upper panel) and secondary (bottom panel) generation. (**C**) Mammospheres self-renewal estimated in WT and TR MCF-7 mammospheres. (**D**) Flow cytometry analysis of CD44 and CD24 expression in secondary WT and TR MCF-7 mammospheres. Representative flow cytometry data are shown (left panel). The values are indicative of CD44- and CD24-positive cell percentages expressed as fold change (right panel). (**E**) qRT-PCR in WT (left panel) and TR (right panel) MCF-7 mammospheres to analyze CD44 and CD24 mRNA levels. 18S mRNA was used to normalize the variability in template loading. The histograms represent the means ± S.D. of three different experiments each performed in triplicate. (**F**) Representative plots of flow cytometry (left panel) and related quantification (right panel) of ALDH1^+^ cells. The histograms represent the mean of ± S.D. of three separate experiments. Data were analyzed by Student’s T test using the GraphPad Prism 7 software program. * *p* < 0.05 vs C; ** *p* < 0.01 vs. C.

To investigate the specific role of low adiponectin levels in influencing BCSC behavior in the context of hormone resistance, we first analyzed mammosphere formation from WT and TR MCF-7 cells in the presence or absence of adipokine (Figure 1A). In this experimental model, cells are compelled to survive and proliferate under anchorage-independent conditions, forming floating spherical colonies called mammospheres, which allow for the enrichment, proliferation, and characterization of breast cancer cells with a stem-like phenotype [45]. The results of our experiments demonstrated that in tamoxifen resistance conditions, adiponectin increased the MFE of the secondary generation to a greater extent than that observed in the primary generation (Figure 1B). In contrast, in hormone-responsive cells, adiponectin was unable to induce a significant increase in MFE (Figure 1B). These findings were consistent with the observation that TR MCF-7 cells exhibited a greater capability for self-renewal compared to WT MCF-7 cells, which was further enhanced upon adiponectin treatment (Figure 1C). The assessment of characteristic cell surface marker expression was carried out to detect the enrichment of cancer stem cells (CSCs) within mammospheres. Interestingly, adiponectin-treated TR MCF-7 cells obtained from the secondary generation of mammospheres revealed the overexpression of the transmembrane glycoprotein CD44 and the downregulation of the cell membrane sialoglycoprotein CD24 (CD44^+^/CD24^−^), as evidenced by flow cytometry and confirmed by qRT-PCR analysis (Figure 1D,E). Moreover, a significantly increased expression of aldehyde dehydrogenase-1 (ALDH1), another marker of stemness, was observed in adiponectin-treated TR MCF-7 cells grown as mammospheres (Figure 1F). On the contrary, in WT MCF-7 mammospheres, a reduction in the ALDH1^+^ subpopulation was detected (Figure 1F). It is worth noting that high levels of ALDH1 are generally associated with an increased risk of metastasis, poorer prognosis, and unfavorable clinical outcomes in breast cancer patients [46].

**Figure 2:**
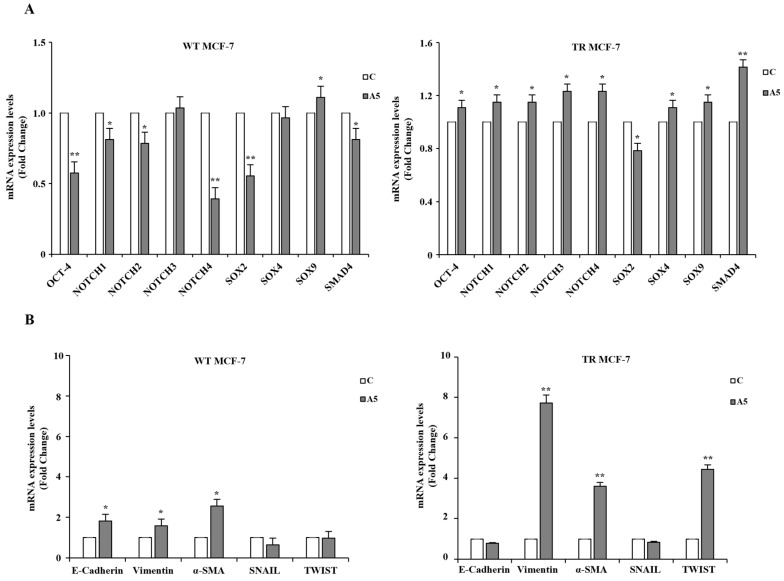
Cancer stem-like phenotype identification according to stem cell and EMT marker profiling in MCF-7 mammospheres. (**A**) qRT-PCR of genes involved in stemness in secondary WT (left panel) and TR (right panel) MCF-7 mammospheres, untreated (control, C) or treated with adiponectin 5 μg/mL (A5). (**B**) Relative expression of mRNAs encoding epithelial (E-cadherin) and mesenchymal (Vimentin, α-SMA, SNAIL, and TWIST) markers evaluated by qRT-PCR in WT (left panel) and TR (right panel) MCF-7 mammospheres. 18S mRNA was used to normalize the variability in template loading. The histograms represent the means ± S.D. of three different experiments each performed in triplicate. Data were analyzed by Student’s *T* test using the GraphPad Prism 7 software program. * *p* < 0.05 vs. C; ** *p* < 0.01 vs. C.

Further evidence supporting the enrichment of stem-like cells in adiponectin-treated TR MCF-7 mammospheres was provided by the high expression levels of genes associated with the maintenance of stemness, such as Oct-4, Notch (isoforms 1, 2, 3, 4), Sox (isoforms 2, 4, 9) and SMAD4 (Figure 2A). In addition, adiponectin treatment resulted in a reduction in E-cadherin gene levels, with a concomitant increase in the expression of mesenchymal markers, including vimentin, α-SMA, and TWIST (Figure 2B), addressing a switch into the epithelial-to-mesenchymal transition (EMT) program in hormone-resistant cells. On the contrary, an increase in E-cadherin expression was observed in adiponectin-treated WT MCF-7 mammospheres (Figure 2B). Taken together, these results suggest that low levels of adiponectin may stimulate CSC activity, particularly in hormone-resistant breast cancer cells.

### 3.2. Proteomic and GO Enrichment Analysis of Differentially Expressed Proteins (DEPs) in MCF-7 Mammospheres

**Figure 3:**
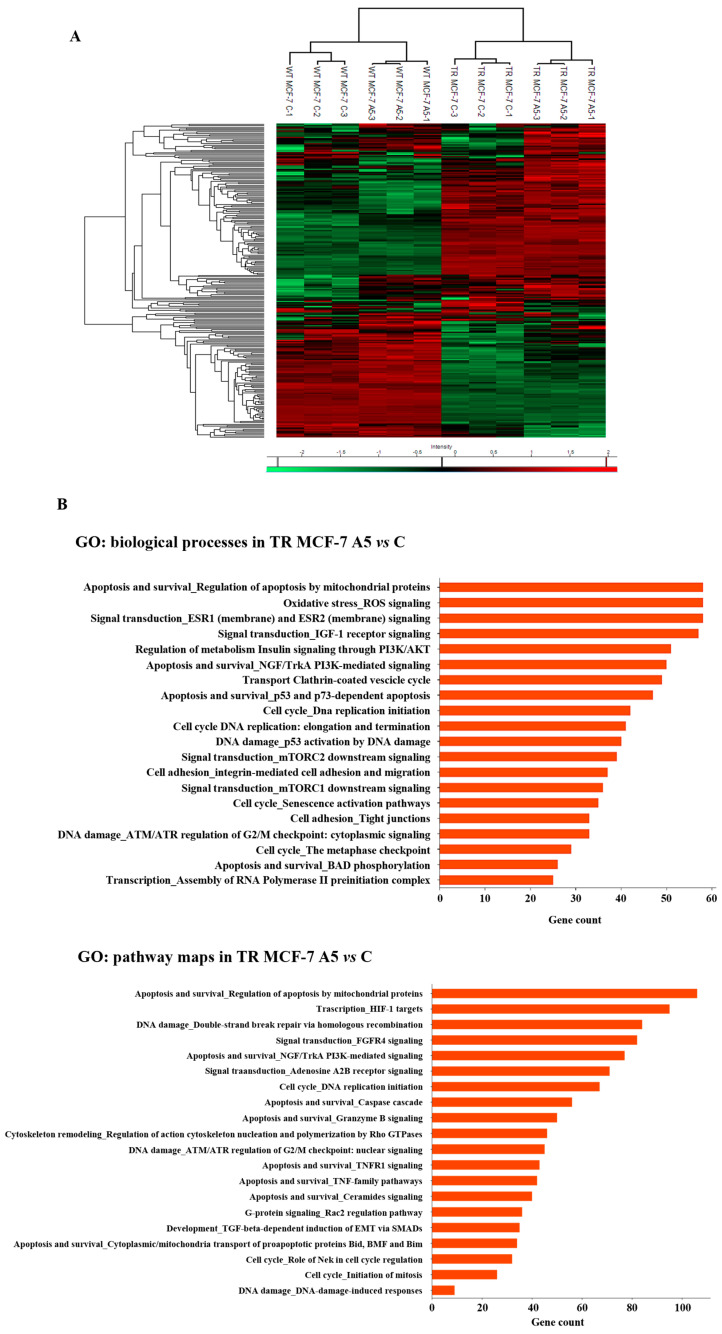
Effects of adiponectin on proteomic expression in WT and TR MCF-7 mammospheres. Statistical analysis on the nLC-MS/MS data was performed by Perseus software. (**A**) Hierarchical clustering and heat map of protein intensity in WT and TR MCF-7 mammospheres, untreated (control, C) or treated with adiponectin 5 µg/mL (A5), are shown. Normalization was based on z-scores and the data for each row were normalized by the mean value calculated for the same row. The intensity value depicts the direction of protein expression as upregulated (red) or downregulated (green). Three repeats per condition were performed. (**B**) The top 20 GO biological processes (upper panel) and pathway maps (bottom panel) terms with the highest statistical significance for DEPs in TR MCF-7 mammospheres. The vertical axis represents the GO biological processes and pathway map terms, and the horizontal axis represents the number of genes.

The modulatory role of adiponectin in the proteome profile of WT and TR MCF-7 mammospheres was investigated by label-free liquid chromatography–tandem mass spectrometry (LC-MS/MS). The analysis of three different biological replicates identified 6283 proteins at a protein false discovery rate (FDR) of <1%. Hierarchical clustering, carried out based on protein intensity, highlighted that the identified DEPs can serve as a proteomic signature for distinguishing adiponectin-treated cells vs. control, as well as hormone-responsive vs. hormone-resistant breast cancer mammospheres, as shown by heat map (Figure 3A). Next, we performed GO enrichment analysis of the identified DEPs to gain insights into the biological processes and pathway maps that are affected in the adiponectin-treated TR MCF-7 mammospheres compared to control group by using MetaCore^TM^ software (Clarivate Analytics), a widely known database for protein–protein signaling. In hormone-resistant mammospheres, which exhibited an augmented adiponectin-induced BCSC subpopulation, we found that DEPs were significantly enriched with the first 20 GO categories linking to cell cycle progression and apoptosis (Figure 3B).

### 3.3. Impact of Low Adiponectin Levels on Cell Cycle Regulation in TR MCF-7 Mammospheres

**Figure 4:**
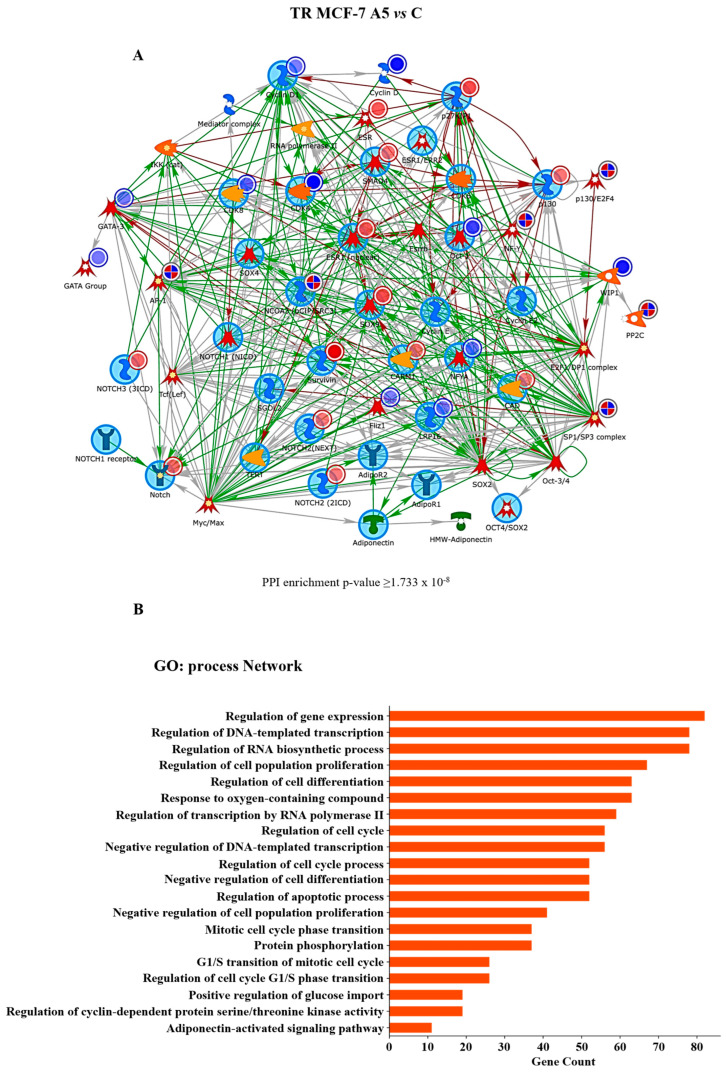
MetaCore^TM^ analysis of protein interactions. Protein–protein interaction network for DEPs in TR MCF-7 cells obtained from adiponectin-treated secondary-generation mammospheres compared to untreated cells (control, C). (**A**) MetaCore^TM^ analysis of the network showing the Adiponectin Receptor 1 (AdipoR1)-Notch-Cyclin D1 (*p*-value > 1.733 × 10^−8^) interaction. Blue circles indicate downregulated proteins, while red circles mark upregulated proteins. (**B**) Enrichment analysis using the most significant GO annotations according to biological processes. The bar graph displays the significant biological processes of the hub proteins present in the network.

It is frequently observed that cell cycle progression is deregulated in breast cancer cells with a stem-like phenotype. This feature enables CSCs to play a pivotal role in tumor initiation, progression, therapeutic resistance, and disease recurrence [47,48]. Based on this evidence and the results of our GO enrichment analysis, we first explored the closest and most central links between endocrine resistance and cell cycle progression regulated by low adiponectin levels, using the Direct Interaction algorithm. Interestingly, network analysis highlighted a significant interaction between proteins related to cell cycle regulation, such as Cyclin D1, p27kip1, CDK4, CDK8, and those correlated to stem cell activity, including Notch family, SOX9, SMAD4, GATA group, in adiponectin-treated TR MCF-7 mammospheres (Figure 4A,B).

**Figure 5:**
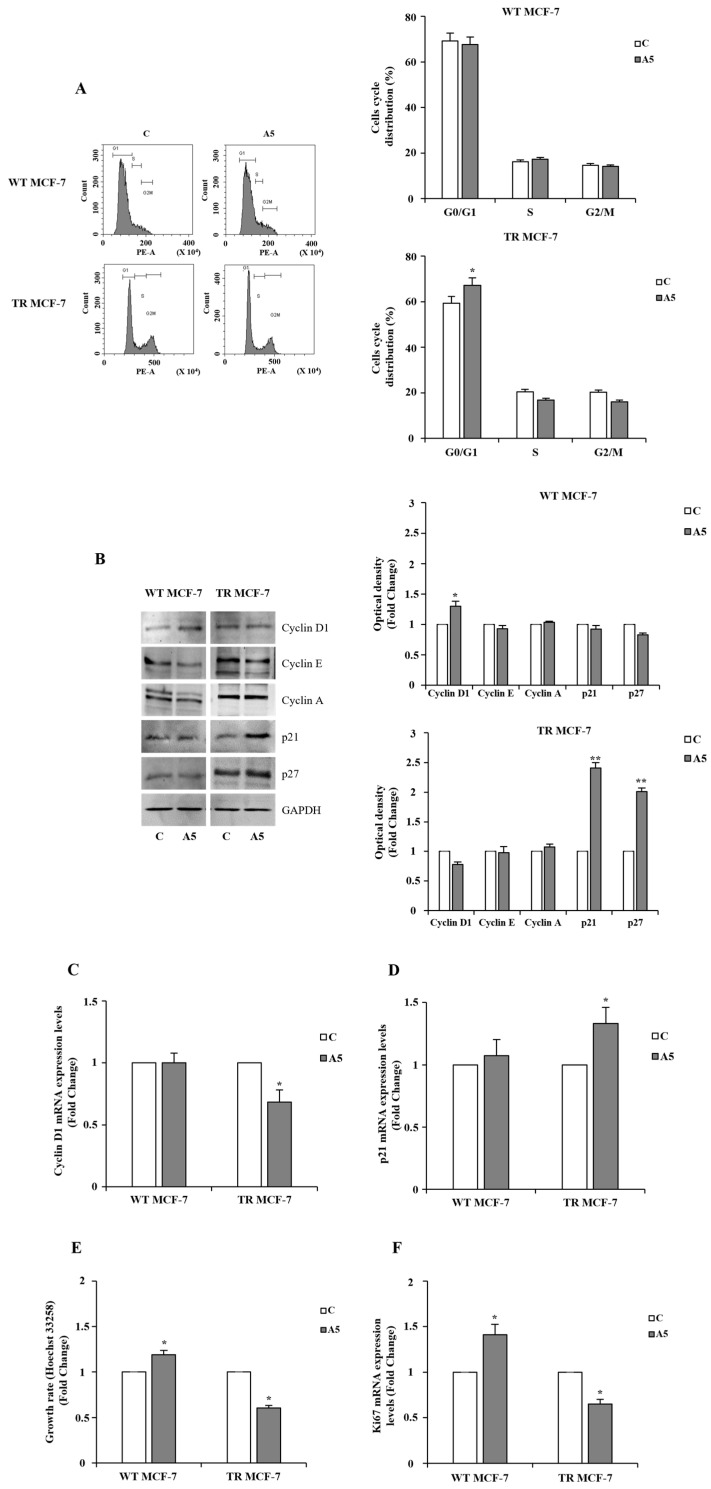
Cell cycle progression and proliferation in adiponectin-treated WT and TR MCF-7 mammospheres. (**A**) Representative flow cytometry plots (left panel) of cell cycle distribution in WT and TR MCF-7 secondary-generation mammosphere-derived cells, untreated (control, C) or treated with adiponectin 5 μg/mL (A5). Cell quantitation in cell cycle phases (right panel). (**B**) Western blotting of Cyclin D1, Cyclin E, Cyclin A, and p21 and p27 expression in WT and TR MCF-7 secondary-generation mammosphere-derived cells. GAPDH was used as loading control. Images are representative of three different experiments (left panel) performed with three independent biological replicates. The histograms represent the means ± S.D. of three different experiments in which band intensities were evaluated in terms of optical density arbitrary units and expressed as fold changes in C, assumed to be 1 (right panel). Relative expression of mRNA levels of Cyclin D1 (**C**) and p21 (**D**) analyzed by qRT-PCR. 18S mRNA was used to normalize the variability in template loading. (**E**) Growth rate in mammosphere-derived WT and TR MCF-7 cells assessed by FluoReporter Blue Fluorometric dsDNA Quantitation Kit. Cell proliferation was expressed as fold change. (**F**) qRT-PCR for Ki67 mRNA expression. 18S mRNA was used to normalize the variability in template loading. The histograms represent the means ± S.D. of three different experiments each performed in triplicate. Data were analyzed by Student’s T test using the GraphPad Prism 7 software program. * *p* < 0.05 vs. C; ** *p* < 0.01 vs. C.

Next, to explore the effects of adiponectin on cell cycle phases in BCSCs, FACS analysis was performed. In TR MCF-7 cells, adiponectin induced a cell cycle arrest in G0/G1 phase, which was not noticeable in WT MCF-7 cells (Figure 5A). To deeply investigate how adiponectin affects the progression of cell cycle phases in WT and TR MCF-7 mammospheres, the impact of this adipokine on the expression of cell cycle-related proteins was analyzed. The data showed that adiponectin was unable to induce an upregulation of Cyclin D1, Cyclin E, and Cyclin A in hormone-resistant cells (Figure 5B). Moreover, adiponectin treatment resulted in an increased expression of p21 and p27, two cyclin-dependent kinase inhibitors involved in the regulation of the cell cycle, in TR MCF-7 mammospheres, but this was not observed in WT MCF-7 cells (Figure 5B). G0/G1 cell cycle arrest was also supported by the assessment of the mRNA expression levels of two key regulators of the S phase transition, Cyclin D1 and p21, which were downregulated and upregulated, respectively (Figure 5C,D). Quantification analysis of DEPs from proteomic profile confirmed the expression pattern of proteins favoring cell cycle arrest in G0/G1 phase upon adiponectin exposure in hormone-resistant cells (Figure S1). Interestingly, the adiponectin-regulated proteins observed in BCSCs derived from tamoxifen-resistant mammospheres were consistent with their slower proliferation rate (Figure 5E). This was correlated with decreased Ki67 mRNA levels (Figure 5F), which have previously been reported in BCSCs as a worse prognosis marker [49]. On the contrary, an increased growth rate was evidenced in WT MCF-7 cells derived from mammospheres, sustained by high Ki67 levels (Figure 5E,F).

### 3.4. Events Converging on Apoptosis Inhibition in TR MCF-7 Cells upon Adiponectin Exposure

**Figure 6:**
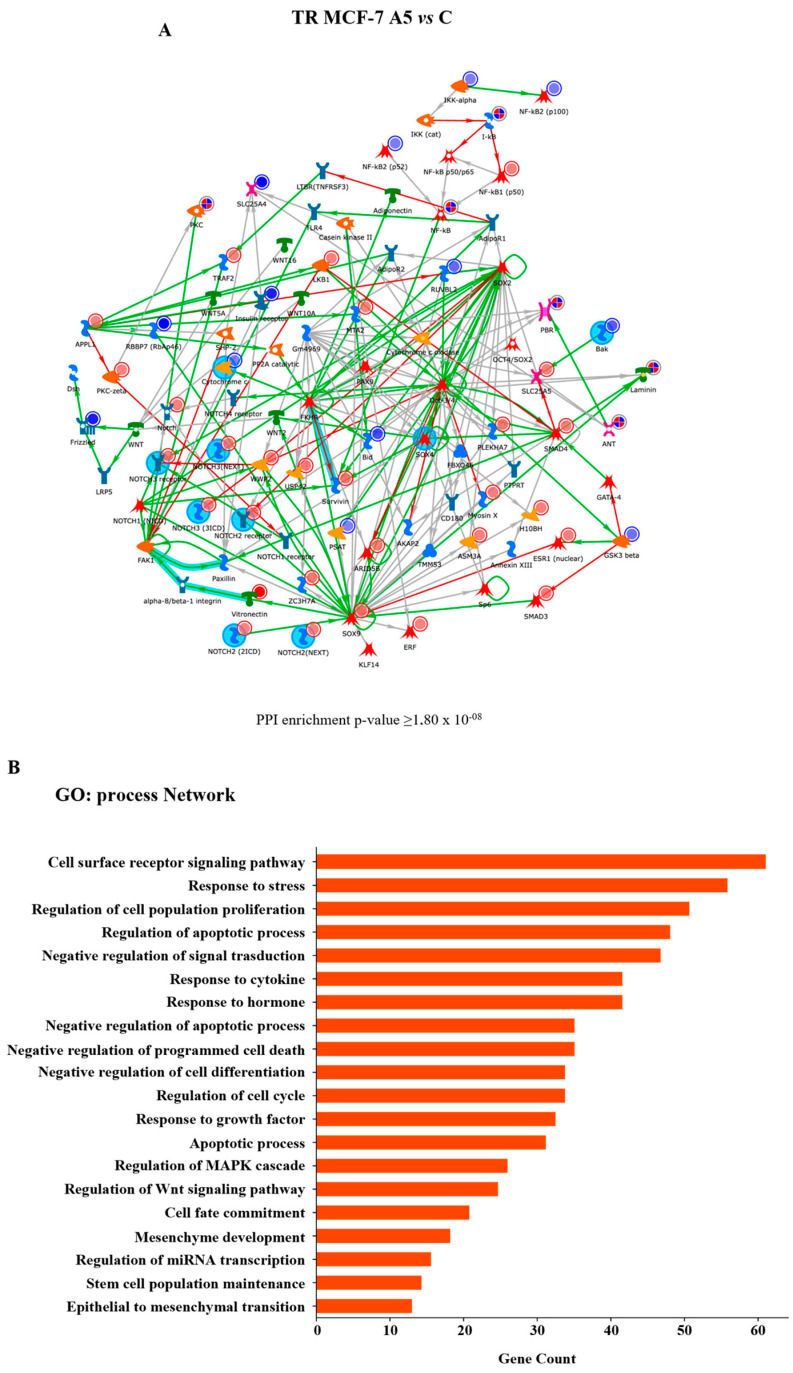
MetaCore^TM^ interaction network analysis. Protein–protein interaction network for DEPs in TR MCF-7 cells obtained from adiponectin-treated secondary-generation mammospheres compared to untreated cells (control, C). (**A**) MetaCore^TM^ analysis of the network evidencing the BID-Notch-AdipoR1 interaction (*p*-value > 1.80 × 10^−8^). Blue circles indicate downregulated proteins, while red circles mark upregulated proteins. (**B**) Enrichment analysis using the most significant GO annotations according to biological processes. The bar graph shows the significant biological processes of the hub proteins present in the network.

Another important mechanism through which breast cancer cells with a stem-like phenotype contribute to endocrine resistance is represented by their capacity to escape from apoptosis [50]. Starting from this observation, the MetaCore^TM^ Direct Interaction algorithm was used to evaluate the possible link between stemness and apoptosis in tamoxifen-resistant cells grown as mammospheres in the presence of low adiponectin levels. Network analysis highlighted a significant interaction between the same DEPs involved in stemness maintenance, as shown above (Figure 4A), and proteins engaged in anti-apoptotic events, such as Bak, Bid, Cytochrome C, and Survivin (Figure 6A,B), as confirmed by the quantification analysis, showing a downregulation of CAPN2 and Bid (Figure S2).

**Figure 7:**
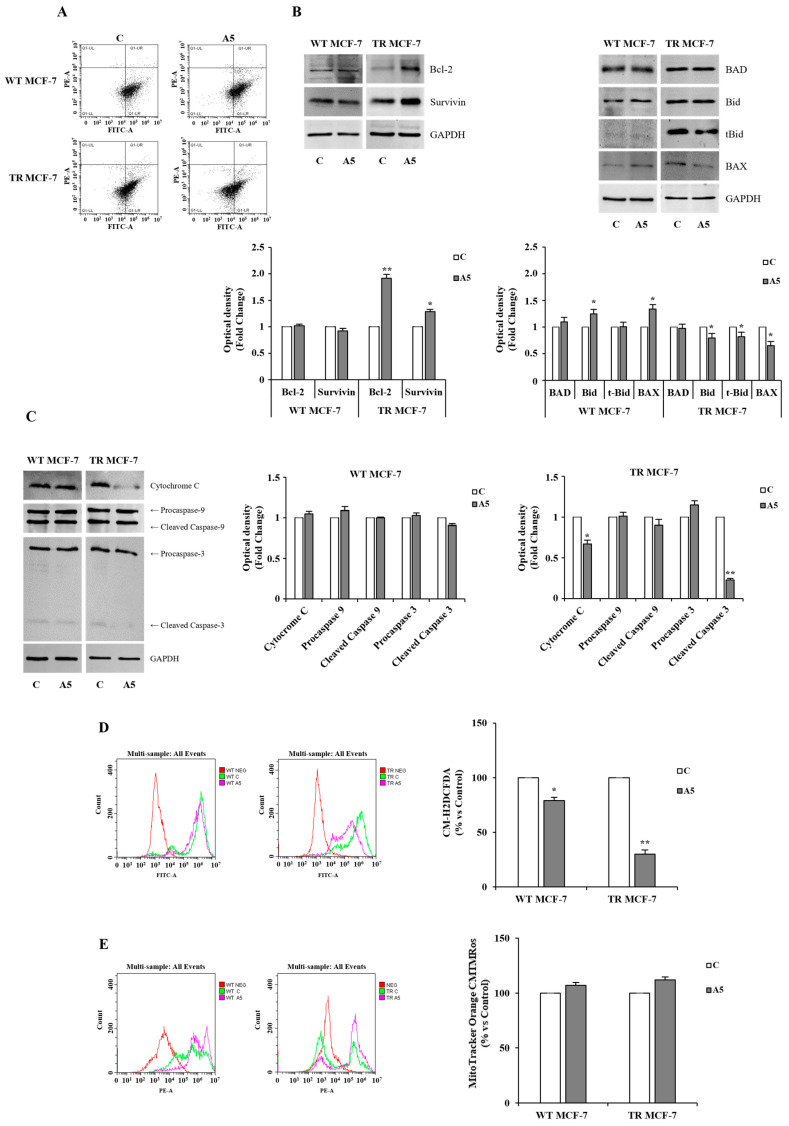
Effects of adiponectin on apoptosis events in TR MCF-7 mammospheres. (**A**) Flow cytometric analysis of secondary-generation WT and TR MCF-7 mammospheres, untreated (control, C) or treated with adiponectin 5 μg/mL (A5) and stained with Annexin V-FITCH and propidium iodide (PI). The four quadrants represent living cells (lower left, Annexin V^−^/PI^−^) and early apoptotic (lower right, Annexin V^+^/PI^−^), late apoptosis (upper right Annexin V^+^/PI^+^), and necrotic (upper left Annexin V^−^/PI^+^) stages. The graphic is representative of three different experiments. Representative images of Bcl-2, Survivin, BAD, Bid, tBid, and Bax ((**B**), upper panel) and Cytochrome C, Caspase-9, and Caspase-3 ((**C**), left panel) expression levels evaluated by Western blotting in protein extracts from secondary generation mammospheres. The images are representative of three different experiments performed with three independent biological replicates. The histograms represent the means ± S.D. of three different experiments in which band intensities were evaluated in terms of optical density arbitrary units and expressed as fold change in C, assumed to be 1 ((**B**), bottom panel and **C**, right panel). GAPDH was used as loading control. Representative flow cytometry overlay images of ROS levels ((**D**), left panel) and mitochondrial membrane potential ((**E**), left panel). Untreated cells were used as negative control. Data are shown as probe fluorescence normalized on viable cell number and expressed as percentage vs. C ((**D**,**E**), right panel). Data were analyzed by Student’s T test using the GraphPad Prism 7 software program. * *p* < 0.05 vs. C, ** *p* < 0.01 vs. C.

According to these data, a significant reduction in apoptotic events was highlighted by Annexin V assay in TR MCF-7 mammospheres following adiponectin treatment (Figure 7A). The anti-apoptotic effects of adiponectin on hormone-resistant BCSCs were also supported by the upregulation of the anti-apoptotic proteins Bcl-2 and Survivin and the downregulation of the pro-apoptotic molecules Bid, tBid, and Bax (Figure 7B). In contrast, all this was not noticeable in the adiponectin-treated WT counterpart (Figure 7A,B). Furthermore, a reduced mitochondrial release of cytochrome C, with consequent decreased cleavage of Caspase-9 and -3, worked to sustain the escape from apoptosis promoted by adiponectin in TR MCF-7 mammospheres BCSCs-enriched (Figure 7C). In addition, adiponectin caused a reduction in intracellular ROS levels (Figure 7D), which are known to be a further BCSC feature correlated to endocrine resistance and escape from apoptosis [48,51]. This could be due to the preserved mitochondrial membrane potential, which prevents the release of cytochrome C and thus apoptotic events in adiponectin-treated TR MCF-7 mammospheres (Figure 7E). Thus, our findings demonstrated that adiponectin, by preventing apoptosis, may contribute to the maintenance of the stem-like phenotype in hormone-resistant BCSCs.

## 4. Discussion

The development and growth of mammary tumors are significantly influenced by the interactions between components of the breast microenvironment, including adipocytes, mesenchymal stem cells, tumor-associated fibroblasts, immune cells, endothelial cells, and malignant cells. These functional interactions give rise to a cancer niche that affects the phenotype of neoplastic cells and contribute to drug resistance, cancer recurrence, and metastasis [52].

Adipocytes are of growing interest as a focal point for the interaction between tumor and stroma. Breast cancer cells promote the dedifferentiation of tumor-adjacent adipocytes, inducing changes in their number and size, together with dysregulated growth factor and pro-inflammatory cytokine secretion, making a more favorable microenvironment to support tumor growth and progression [25,53]. Several reports have investigated the involvement of adipokines in breast cancer progression and recurrence, also highlighting the important role of adiponectin in obesity-related breast cancer [28,29,30,37,38,39,40,54].

Adiponectin, the most abundant fat-derived hormone, is secreted at lower levels in obese individuals, playing a relevant role in ERα-positive breast cancer [27,31,54]. ERα is present in 70% of breast cancer patients and provides the rationale for endocrine therapy aimed at antagonizing ERα signaling and reducing local estrogen production that sustains tumor growth [55]. However, it is well known that a significant proportion of patients present de novo or develop acquired resistance to endocrine therapy [56]. In this context, several reports have documented the involvement of adipokines in potentiating ERα transactivation and in increasing local estrogen production as events determining endocrine therapy failure [57]. Interestingly, in vitro and in vivo studies have shown that low levels of adiponectin stimulate the growth and progression of ERα-positive breast cancer [28,29,37,38,39]. All this occurs through the activation of the mitogen-activated protein kinase (MAPK) pathway, which is responsible for ERα transactivation and Cyclin D1 upregulation, resulting in the stimulation of breast cancer cell growth [39,40]. Thus, the sequence of these events highlights the role of adiponectin at low levels as a growth factor in ERα-positive breast cancer patients.

Among the various microenvironmental cues that can induce hormone resistance in breast cancer, those that stimulate tumor stemness are of particular importance [6,47,58,59].

In the present study, we demonstrated that adiponectin plays a critical role in the maintenance of BCSC bulk in an experimental model of acquired resistance to tamoxifen. For instance, adiponectin exposure was able to enrich tamoxifen-resistant mammospheres in the secondary generation of stem cells, which exhibited a CD44^+^/CD24^−^/ALDH1^+^ phenotype together with increased expression of transmembrane receptors (Notch1, Notch2, Notch3, and Notch4), and transcription factors, including Oct-4, SMAD4, and members of the SOX family (SOX4 and SOX9). Particularly, Notch4 was strongly upregulated in TR MCF-7 cells upon exposure to adiponectin, whereas no noticeable changes were observed in WT MCF-7 cells. The enhanced expression of genes inducing breast cancer cell stemness upon adiponectin exposure in TR mammospheres is related to previous findings demonstrating the relationship between stemness and endocrine resistance.

For instance, it has been reported, in a preclinical setting, that an inhibitor of Notch signaling also suppressed the expression of key genes promoting stemness, such as SOX2, which thwarts BCSC proliferation following endocrine therapy [60]. The role of Notch4 activity in inducing endocrine resistance and the stem cell phenotype was confirmed when previous authors analyzed the loss–gain-of-function phenotypes for Notch4-ICD in MCF-7 cells [61]. The same authors showed how the genomic disruption of Notch4 using a CRISPR approach led to a loss of protein expression and a significant reduction in MFE and ALDH1^+^ cells, especially after tamoxifen and fulvestrant treatment. In contrast, overexpression of Notch4-ICD or its ligand JAG1 confirmed tamoxifen and fulvestrant resistance in parental MCF-7 cells [61]. In addition, using a Patient-Derived Xenograft (PDX), the increased expression of Notch4 was shown to be as a predictive marker of resistance to endocrine treatment. In particular, the study demonstrated the ability of the Notch4 inhibitor RO4929097 to reverse the acquisition of resistance to tamoxifen and fulvestrant treatment in breast cancer cells dissociated from PDX after long-term treatment with antiestrogens [61]. Thus, the authors recommended that endocrine therapy should be combined with drug inhibitors of stemness [61].

It is worth mentioning that an increased expression of mesenchymal markers such as Vimentin, αSMA, SNAIL and TWIST was observed in adiponectin-treated TR MCF-7 cells, addressing the activation of the EMT program in breast cancer cells, which sustains the generation of hormone-resistant breast cancer stem cells, as previously documented [62,63]. In addition, upon exposure to adiponectin, tamoxifen-resistant cells exhibited a slow cycling status, related to low Ki67 expression, decreased Cyclin D1 levels, and increased p21 and p27 contents, all of which are events associated with the acquisition of a quiescent status [49]. The capacity of BCSCs to persist in the G0/G1 phase of the cell cycle is of particular significance, as it confers resistance to damage from chemotherapy and radiotherapy [64]. This highlights the role of low adiponectin levels in promoting a more aggressive phenotype in patients with ER-positive breast cancer who are developing resistance to tamoxifen therapy. Finally, the escape from apoptosis is another important mechanism through which BCSCs contribute to endocrine resistance [50,65]. Interestingly, in TR MCF-7 cells derived from mammospheres treated with adiponectin, an increased expression of anti-apoptotic proteins occurred, concomitant with reduced levels of ROS [51]. The preserved mitochondrial membrane potential contributes to preventing the cytosolic release of the pro-apoptotic factor, Cytochrome C, favoring the acquisition of anti-apoptotic features of stem cells present in TR MCF-7 mammospheres upon adiponectin exposure. Proteomic analysis, through GO of biological processes, pathway maps, and process networks, further evidenced the different strategies through which adiponectin maintain the cancer stem phenotype bulk in TR mammospheres.

## 5. Conclusions

The development of hormone resistance during endocrine therapy represents a significant challenge in the management of ERα-positive breast cancer. Over time, numerous cellular mechanisms have been proposed to explain this phenomenon, among which the role of BCSCs has attracted particular attention. Many studies have identified adipocytes as crucial regulators of the biological behavior of BCSCs in the TME. Interestingly, low adiponectin levels, secreted by dysfunctional obesity-related adipocytes, have been found to contribute to a more aggressive tumor phenotype.

The findings of this study demonstrated that low levels of adiponectin play a role in the acquisition of several stem-like characteristics in tamoxifen-resistant cells. Our results highlighted that cell cycle arrest in G0/G1 phase, characterized by p21^high^, p27^high^ and ki67^low^, together with the ability to escape from apoptosis are key mechanisms by which adiponectin facilitates the maintenance of cancer stem cell bulk in an acquired tamoxifen-resistant model. All this suggests that low levels of adiponectin promote endocrine resistance through the enrichment of a pool of BCSCs in a quiescent status in obese breast cancer patients.

While these results were limited to an in vitro 3D cellular model, they address the important role exerted by low adiponectin levels in the acquisition of a more endocrine resistant ERα-positive breast cancer phenotype.

Future and deeper in vitro and in vivo studies examining the contribution of the BCSC subpopulation and the regulation of signaling pathways involved in the survival and maintenance of the CSC niche could lead to the design of personalized therapies for the treatment of breast cancer.

## Figures and Tables

**Table 1:** List of primers used.

OLIGO NAME	SEQUENCE 5′-3′
ALPHA-SMA	Fw-AGACATCAGGGGGTGATGGT
	Rw-CATGGCTGGGACATTGAAAG
CD24	Fw-GCTCCTACCCACGCAGATT
	Rw-GTGAGACCACGAAGAGACTGG
CD44	Fw-GAAGAAGGTGTGGGCAGAAGA
	Rw-ACCATTTCCTGAGACTTGCTG
CYCLIN D1	Fw-GATGCCAACCTCCTCAACGAC
	Rw-CTCCTCGCACTTCTGTTCCTC
E-CADHERIN	Fw-TGCCCAGAAAATGAAAAAGG
	Rw-GTGTATGTGGCAATGCGTTC
NOTCH1	Fw-GTGACTGCTCCCTCAACTTCAAT
	Rw-CTGTCACAGTGGCCGTCACT
NOTCH2	Fw-CACCCCAGCTGCTACTCACA
	Rw-GCCAACCCAGCCTGCAT
NOTCH3	Fw-CCTGTCTTCCTGGGTTTGAG
	Rw-CAGAACTGGCCTGTGCACTC
NOTCH4	Fw-CCAACCCTGCGATAATGCGAG
	Rw-AGTCATCCGTTGAGACCCTGC
OCT-4	Fw-AGCGACTATGCACAACGAGA
	Rw-CCATAGCCTGGGTACCAAA
p21	Fw-GCATGACAGATTTCTACCACTCC
	Rw-AAGATGTAGAGCGGGCCTTT
SMAD4	Fw-GGAGCTCATCCTAGTAAATG
	Rw-GACGGGCATAGATCACATGA
SNAIL	Fw-CGAGTGGTTCTTCTGCGCTA
	Rw-GGGCTGCTGGAAGGTAAACT
SOX2	Fw-CACATGAAGGAGCACCCGGATTAT
	Rw-GTTCATGTGCGCGTAACTGTCCAT
SOX4	Fw-GGCCTCGAGCTGGGAATCGC
	Rw-GCCCACTCGGGGTCTTGCAC
SOX9	Fw-AACGCCGAGCTCAGCAAGA
	Rw-TTCTTGTGCTGCACGCGCA
TWIST	Fw-TCCAAATTCAAAGAAACAGGGCG
	Rw-CAGAATGCAGAGGTGTGAGGA
VIMENTIN	Fw-GAGAACTTTGCCGTTGAAGC
	Rw-GCTTCCTGTAGGTGGCAATC
Ki67	Fw-TCCTTTGGTGGGCACCTAAGACCTG
	Rw-TGATGGTTGAGGTCGTTCCTTGATG
18S	Fw-CGGCGACGACCCATTCGAAC
	Rw-GAATCGAACCCTGATTCCCCGTC

## Data Availability

The data presented in this study are available on request from the corresponding author. The mass spectrometry proteomics data have been deposited to the ProteomeXchange Consortium via the PRIDE partner repository with the dataset identifier PXD059964.

## Supplementary Materials

The following supporting information can be downloaded at https://www.mdpi.com/article/10.3390/cells14040286/s1, Figure S1: Quantification of DEPs from proteomic analysis. Box plots show cell cycle-regulating protein expression in WT and TR MCF-7 mammospheres, untreated (Control, C) or treated with adiponectin 5 µg/mL (A5). Data were analyzed by ordinary one-way ANOVA test using the GraphPad Prism 7 software program. An adjusted *p*-value cutoff ≤ 0.05 and log fold change cutoff ≥ 1 have been applied to determine significantly regulated proteins in each pairwise comparison by default. Figure S2: Quantification of apoptosis-related DEPs evidenced by proteomic analysis. Box plots show pro-apoptotic protein expression in WT and TR MCF-7 mammospheres, untreated (Control, C) or treated with adiponectin 5 µg/mL (A5). Data were analyzed by ordinary one-way ANOVA test using the GraphPad Prism 7 software program. An adjusted *p*-value cutoff ≤ 0.05 and log fold change cutoff ≥1 have been applied to determine significantly regulated proteins in each pairwise comparison by default.

## Abbreviations

The following abbreviations are used in this manuscript:

ER	Estrogen receptor
TAM	Tamoxifen
BCSCs	Breast cancer stem Cells
EMT	Epithelial-to-mesenchymal transition
TME	Tumor microenvironment
WT MCF-7	Hormone-responsive MCF-7 breast cancer cells
TR MCF-7	Tamoxifen-resistant MCF-7 breast cancer cells
MFE	Mammosphere-forming efficiency
qRT-PCR	Quantitative real-time reverse transcription PCR
ROS	Reactive oxygen species
PAC	Protein Aggregation Capture
DTT	Dithiothreitol
ACN	Acetonitrile
TEAB	Triethylammonium bicarbonate
FA	Formic acid
LC-MS/MS	Liquid chromatography–tandem mass spectrometry
MS	Mass spectrometry
DIA	Data Independent Acquisition
CSCs	Cancer stem cells
ALDH1	Aldehyde dehydrogenase 1
DEPs	Differentially expressed proteins (DEPs)
