# Supplementary material for: Adiponectin Influences the Behavior of Stem Cells in Hormone-Resistant Breast Cancer
Source: Cells. 2025 Feb 15;14(4):286. doi: 10.3390/cells14040286 (PMC11853953; doi:10.3390/cells14040286)
Supplement: Supplementary file 1 [file cells-14-00286-s001.zip › cells-3464594-supplementary.pdf]

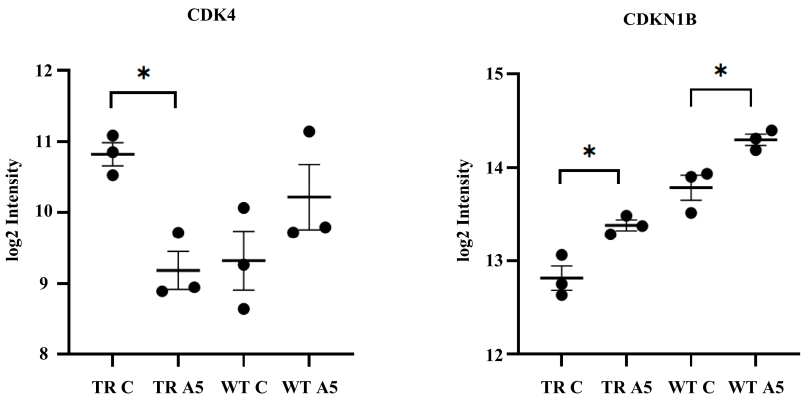

Figure S1: Quantification of DEPs from proteomic analysis. Box plots show cell cycle-regulating protein expression in WT and TR MCF-7 mammospheres, untreated (Control, C) or treated with adiponectin 5 µg/mL (A5). Data were analyzed by ordinary one-way ANOVA test using the GraphPad Prism 7 software program. An adjusted *p*-value cutoff $\leqslant$ 0.05 and log fold change cutoff $\geqslant$1 have been applied to determine significantly regulated proteins in each pairwise comparison by default.

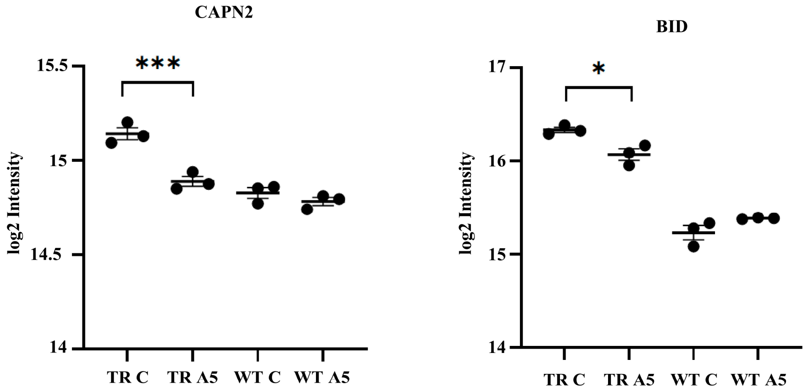

Figure S2: Quantification of apoptosis-related DEPs evidenced by proteomic analysis. Box plots show pro-apoptotic protein expression in WT and TR MCF-7 mammospheres, untreated (Control, C) or treated with adiponectin 5 µg/mL (A5). Data were analyzed by ordinary one-way ANOVA test using the GraphPad Prism 7 software program. An adjusted *p*-value cutoff $\leq$ 0.05 and log fold change cutoff $\geq$1 have been applied to determine significantly regulated proteins in each pairwise comparison by default.
